# Health expenditure: how much is spent on health and care worker remuneration? An analysis of 33 low- and middle-income African countries

**DOI:** 10.1186/s12960-023-00872-y

**Published:** 2023-12-20

**Authors:** Hapsatou Toure, Maria Aranguren Garcia, Juana Paola Bustamante Izquierdo, Seydou Coulibaly, Benjamin Nganda, Pascal Zurn

**Affiliations:** 1 Health Systems Governance and Financing Department, World Health Organization, Avenue Appia 20, 1211 Geneva 27, Switzerland; 2 Health Workforce Department, World Health Organization, Avenue Appia 20, 1211 Geneva 27, Switzerland; 3 Inter-Country Support Team for West Africa, World Health Organization, Ouagadougou, Burkina Faso; 4 Inter-Country Support Team for Eastern and Southern Africa, World Health Organization, Harare, Zimbabwe

**Keywords:** Health accounts, Health and care workforce, Remuneration, Africa, WAEMU, ECOWAS, ECCAS, SADC, Government health expenditure

## Abstract

**Objectives:**

To assess the amount spent on health and care workforce (HCW) remuneration in the African countries, its importance as a proportion of country expenditure on health, and government involvement as a funding source.

**Methods:**

Calculations are based on country-produced disaggregated health accounts data from 33 low- and middle-income African countries, disaggregated wherever possible by income and subregional economic group.

**Results:**

Per capita expenditure HCW remuneration averaged US$ 38, or 29% of country health expenditure, mainly coming from domestic public sources (three-fifths). Comparable were the contributions from domestic private sources and external aid, measured at around one-fifth each—23% and 17%, respectively. Spending on HCW remuneration was uneven across the 33 countries, spanning from US$ 3 per capita in Burundi to US$ 295 in South Africa. West African countries, particularly members of the West African Economic and Monetary Union (WAEMU), were lower spenders than countries in the Southern African Development Community (SADC), both in terms of the share of country health expenditure and in terms of government efforts/participation. By income group, HCW remuneration accounted for a quarter of country health expenditure in low-income countries, compared to a third in middle-income countries. Furthermore, an average 55% of government health expenditure is spent on HCW remuneration, across all countries. It was not possible to assess the impact of fragile and vulnerable countries, nor could we draw statistics by type of health occupation.

**Conclusions:**

The results clearly show that the remuneration of the health and care workforce is an important part of government health spending, with half (55%) of government health spending on average devoted to it. Comparing HCW expenditure components allows for identifying stable sources, volatile sources, and their effects on HCW investments over time. Such stocktaking is important, so that countries, WHO, and other relevant agencies can inform necessary policy changes.

## Résumé

**Objectifs:**

Évaluer le montant dépensé pour la rémunération du personnel de santé et de soins dans les pays africains, son importance en tant que proportion de la dépense de santé des pays, et déterminer la contribution des sources de financement, en particulier du gouvernement.

**Méthodes:**

Les calculs sont effectués à partir de données désagrégées des comptes de la santé produites par 33 pays africains à revenu faible et intermédiaire classés par groupe économique sous-régional lorsque possible.

**Résultats:**

La rémunération du personnel de santé et de soins s'élevait en moyenne à 38 dollars par habitant, soit 29% de la dépense de santé des pays, et provenait principalement de sources publiques domestiques—à hauteur des trois-cinquièmes. La participation des sources privées domestiques et de l'aide extérieure était à peu près comparable—estimée à un-cinquième chacune, 23% et 17%, respectivement. Les dépenses consacrées à la rémunération du personnel de santé et de soins étaient inégales dans les 33 pays, allant de 3 dollars par habitant au Burundi à 295 dollars en Afrique du Sud. En fait, les pays d’Afrique de l’Ouest, et plus particulièrement ceux de l’Union économique et monétaire ouest africaine (UEMOA), ont dépensé moins que les pays de la Communauté de développement de l'Afrique australe (SADC), tant en termes de part de la dépense de santé des pays qu’en termes d’efforts/de la participation du gouvernement. Par groupe de revenu, la rémunération du personnel de santé et de soins représentait un quart de la dépense de santé des pays à faible revenu, contre un tiers de celles des pays à revenu intermédiaire. En outre, 55% en moyenne des dépenses publiques de santé, tous pays confondus, sont consacrées à la rémunération du personnel de santé et de soins. Il n'a pas été possible d'évaluer l'impact des pays fragiles et vulnérables, ni de dégager des tendances par type de personnel de santé.

**Conclusions:**

Les résultats montrent clairement que la rémunération du personnel du santé et de soins représente une part importante des dépenses publiques de santé; la moitié en moyenne (55%), dans ce groupe de 33 pays africains à revenu faible et intermédiaire. La comparaison des différentes composantes de la dépense permettra au fil du temps d’identifier les sources stables, les sources volatiles et leurs effets éventuels sur les investissements. Ce type de données est important pour que les pays, l'OMS et les autres institutions intéressées, puissent apporter leur contribution aux réformes politiques qui pourraient s’avérer nécessaires.

**Mots clés:**

Comptes de la santé, personnel de santé et de soins, rémunération, Afrique, UEMOA, CEDEAO, CEEAC, SADC, dépenses publiques de santé.

## Background

A health and care workforce (HCW) of adequate size and competence is critical to achieving the United Nations health-related Sustainable Development Goals (SDG3), Universal Health Coverage (UHC), and health security [1]. Yet globally, most countries face multiple challenges—shortages, suboptimal education and training, deployment, performance, productivity, working conditions, and retention—which, exacerbated by limited investment in health workforce education, affect the availability, accessibility, quality, and performance of national health systems and services, preventing a large majority of the population from accessing the appropriate health services they need. Hence, achieving SDG3 will require strengthening the education, training, recruitment, and equitable deployment of HCWs in a paradigm shift to make investments more efficient, effective, and relevant to country needs [2–4]. Advancing this objective will impact health expenditure, particularly HCW remuneration—which, along with pharmaceuticals, is a major component of countries’ health expenditure [5, 6]. Of particular bearing are low- and middle-income African countries, which are expected to account for half of the 10 million global shortage by 2030 [3, 7].

Within this context, a better understanding of government HCW expenditure is needed to support national policy-makers and relevant global agencies to develop more effective and sustainable investments over time. This paper seeks to better understand the scope of expenditure on HCW remuneration and its funding sources in Africa. The specific objectives are to:

1. assess the level of HCW remuneration expenditure and its relative size as part of countries’ overall health expenditure;
2. ascertain governments’ contribution compared to other funding sources; and
3. weigh in government HCW allocation as part of overall government health expenditure.

Furthermore, disaggregation by income group and relevant subregional economic entity is provided for benchmarking purposes and to inform decision-making.

## Methods

### The System of Health Accounts (SHA) and its boundaries measuring HCW expenditure

We use country-produced health accounts data to assess/discuss the magnitude of HCW expenditure in African countries [8]. In short, health accounts help countries trace each dollar spent on health—from source to use—by measuring the magnitude and flow of expenditure on health-defined services consumed by a country’s resident population over a specified period (generally a year^1^)—regardless of where the money originated (public, private, or external sources^2^) or the location of service provision/consumption (hospitals/health centers). Thus, health accounts measure health expenditure associated with all activities that primarily promote, prevent, treat or rehabilitate people’s health status—e.g., disease prevention/cure in hospitals; home-based care for chronic conditions; or information/education/counseling campaigns in schools, prisons, or enterprises. They also measure expenditures on administering the health system at central and regional levels in decentralized countries. 

The starting point for determining the boundaries of HCW expenditure is the System of Health Accounts (SHA), the international framework measuring country health expenditures [9]. It distinguishes between current-year consumption—i.e., recurrent spending—and spending for developing/acquiring health infrastructure consumed over years—i.e., capital/development spending. The former comprises current expenditure; and the latter, capital expenditure (Box 1). Key is understanding that **pre-service education**—provided to students prior to joining the HCW—is considered capital expenditure, whereas **in-service training**—namely, professional trainings, peer-learning activities, and/or guidance sessions on newly released/updated recommendations for treatment protocols—is considered current health expenditure. This paper only considers the latter.

Furthermore, HCW remuneration is considered in its entirety/complexity in various country contexts. On one hand, this entails wages and social contributions to **salaried workers**—including any benefits/allowances in cash or in kind, e.g., housing, fee waivers, etc. For the latter, the monetary equivalent of in-kind benefits is reported. On the other hand is the income of **self-employed** professionals (Box 2). This study follows the international standards defined as “all people engaged in actions whose primary intent is to enhance health” [10], a definition embracing anyone working—either in a publicly owned facility or as a private practitioner—towards promoting, restoring, or maintaining health. This includes management/support staff—part-time and full—located in health facilities or in communities, e.g., community health workers.

The baseline information was calculated in July 2022 for 2019, the year before the COVID-19 pandemic. Country health accounts reports, broken down by factor of provision (FP), can be retrieved from the Documentation Center of WHO’s Global Health Expenditure Database (GHED) (Box 3). The latest available data before 2019 were used when no data were available for 2019 (Appendix 1). Population size and income group data were extracted from GHED Data Explorer in July 2022 (2021 update) [11]. Four parameters of interest were examined:

1. The share of country health expenditure allocated to HCW remuneration; i.e., the sum of expenditure for compensating employees and paying self-employed professionals, over country health expenditure.
2. The main funding sources’ level of participation in HCW remuneration.
3. How much government health expenditure went to remunerating HCW.
4. Where respective government efforts stand in country comparisons.

Wherever possible, estimates were averaged across countries and by income group or subregional economic group [12–15] as some of them have common economic criteria to respect, which tends to some degree of harmonization in public spending, including spending on the HCW. This may have an impact on policy formulation, since they interact—e.g., within the WAEMU countries, the ratio of government wage bill to tax revenue cannot exceed 35% [16].

Averages are unweighted and all values are in US$, converted from national currency units using the GHED conversion rate expressed in real terms (2019). Country-specific gross domestic product deflators were used to convert current values to constant values.

Box 1: Glossary of terms used It is noteworthy that total health expenditure (THE), as defined by the System of Health Accounts framework, includes both current and capital spending on health. In this article, the terms “health spending” and “country health spending” are used synonymously with “current health expenditure.” Capital expenditure, the other part of THE, is not included. 
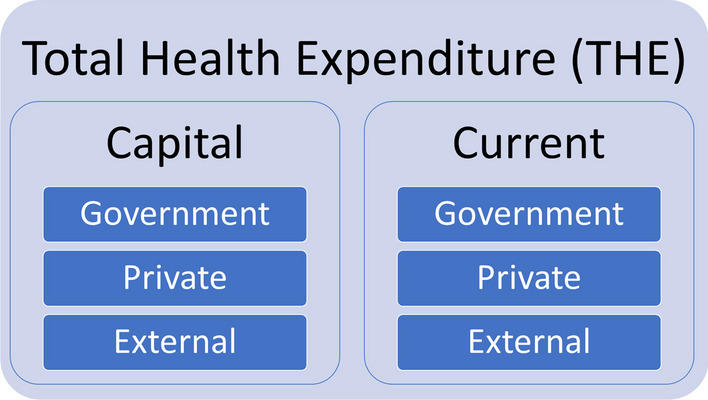

Nevertheless, of importance to both is the composition of the spending. Broadly speaking, funding sources can be grouped into three main groups—government, private, and external. Government and private jointly constitute “domestic spending,” whereas spending from external sources is health spending funded by “non-domestic sources”. The latter corresponds mainly to external aid, including grants and concessional loans to countries from bilateral, multilateral, and private external donors. Recipients of external aid are generally either the government or non-profit institutions, such as non-governmental organizations. Government, on the other hand, refers to spending from its own resources, mobilized though taxation and other revenues, but it also includes spending from all forms of mandatory pre-payment funds—e.g., those generated by contributions to mandatory social health insurance—where they exist. While private spending, the other part of domestic spending, encompasses voluntary health insurance but also spending by locally funded non-profit institutions, employers’ own health services for their workers, and out-of-pocket spending.

Box 2: The System of Health Accounts’ (SHA) Factor of Provision (FP) classification and its categories Factor of provision (FP) classification of the System of Health Accounts (SHA) framework describes the factor inputs used by providers to produce the goods and services consumed or the activities conducted in the system. In simpler terms, they describe the inputs used in producing the health services and goods consumed by the population. They are grouped into five main categories: (i) compensation of employees; (ii) self-employed professional remuneration; (iii) material and services used—among which are pharmaceuticals and other goods and services, such as training; (iv) consumption of fixed capital; and (v) other items of spending on inputs.

**Table:** 

**FP.1**	**Compensation of employees**
FP.1.1	Wages and salaries
FP.1.2	Social contributions
FP.1.3	All Other costs related to employees
**FP.2**	**Self-employed professional remuneration**
**FP.3**	**Materials and services used**
FP.3.1	Health care services
FP.3.2	Health care goods
FP.3.2.1	Pharmaceuticals
FP.3.2.2	Other health care goods
FP.3.3	Non-health care services
FP.3.3.1	Training
FP.3.3.2	Technical Assistance
FP.3.3.3	Operational research
FP.3.3.nec	Other non-health care services, not elsewhere classified
FP.3.4	Non-health care goods
FP.3.nec	Other materials and services used, not elsewhere classified
**FP.4**	**Consumption of fixed capital**
**FP.5**	**Other items of spending on inputs**
**FP.nec**	**Unspecified factors of health care provision, not elsewhere classified**

In this article, HCW remuneration refers to the sum of the first two categories, namely, FP.1 compensation of employees and FP.2 self-employed professional remuneration. However, country teams often struggle to collect specific information on the latter; and 22 countries out of the 33 analyzed have reported an expenditure amount against self-employed professional remuneration. In addition, since the topic is the *remuneration* of the workforce, any expenditure incurred for in-service training—coded FP.3.3.1—is out of scope and hence not part of the calculations. Besides, pre-service education, being capital expenditure, is not taken into consideration—it is recorded under a separated SHA classification, not the factor of provision one.

Box 3: The Global Health Expenditure Database (GHED) WHO’s Global Health Expenditure Database (GHED) is an open-access platform for WHO Member States where countries compare health expenditure data to better understand how they spend money on health and how much different sources contribute—e.g., governments, social health insurance, households, or external aid for health from development partners^3^. Launched 20 years ago to compile internationally comparable data on health expenditure, the database contains health accounts data and WHO-vetted estimates following an annual country consultation process^4^. It is usually accompanied by a report summarizing the data and illuminating key policy issues, thereby stimulating thirst for further investigation/research^5^^,^ 6. By September 2023, the database contained health spending information up to 2020 on > 190 countries.

### WHO’s National Health Workforce Accounts (NHWA)

The National Health Workforce Accounts (NHWA), triggered by the 69^th^ World Health Assembly, is a mechanism for collating/using a set of standardized indicators to generate reliable information/evidence on the health workforce. The objectives are to:

- Enable planning, implementation, and monitoring of HCW policies geared towards UHC.
- Improve comparability of HCW data both nationally and globally.

It is a system by which countries progressively improve the availability, quality, and use of HCW data by annually reporting a set of indicators to support achievement of UHC, SDGs, and other health objectives [17, 18].

The NHWA portal hosts a variety of HCW statistics relevant to measurement. We extracted country medical doctor (MD) density per 10,000 population for the latest year reported (2021 update) [19]. This information was then used gain perspective on countries’ HCW remuneration expenditure.

## Results

*General context*: This paper analyzes current HCW remuneration expenditure in 33 African countries for which disaggregated information was available out of the 47 countries in the WHO African region, as of 30 July 2022. It represents 70% of the 47 countries, 75% of their population (824 million people out of 1 092 billion), 33% of their MDs (180,404), and 80% of their nurses (737,966). Of the world’s population, it comprises 11%, 1.4% of its MDs, and 2.6% of its nurses.

Out of the 33 countries, 16 are low-income and 17 middle-income countries,^7^ representing 76% of low-income and 71% of middle-income countries of the region, respectively (Table 1). By subregional economic group, eight countries are members of the Southern African Development Community (SADC)^8^; six, the West African Economic and Monetary Union (WAEMU); seven, the Economic Community of West African States (ECOWAS) without being members of WAEMU^9^; and seven, the Economic Community of Central African States (ECCAS). Five countries classified as “Other” do not belong to any of the above-mentioned sub-regional groups (Table 2).

**Table 1:** WHO African Region countries by income group

Income group	Country count	Number of WHO Member States by income group	Percent of WHO Member States by income group (%)
Low income	16	21	76
Middle income	17	24	71
Total	33	47	70

The percentages are calculated using the number of countries by income group in the WHO African Region as denominator. The income groups are as per the World Bank 2019 classification of income groups; lower-middle income and upper-middle income groups were merged into a single middle-income group

**Table 2:** WHO African Region countries by subregional economic group

Subregional economic group	Country count	Number of WHO Member States by subregional economic group	Percent of WHO Member States by subregional economic group (%)
ECOWAS	7	7	100
WAEMU	6	8	75
ECCAS	7	11	64
SADC	8	16	50
Other	5	5	100
Total	33	47	70

The percentages are calculated using the number of countries by subregional economic group as denominator. ECOWAS, WAEMU, ECCAS, and SADC, respectively, stand for the Economic Community of West African States, the West African Economic and Monetary Union, the Economic Community of Central African States, and the Southern African Development Community. ECOWAS excludes countries that are members of WAEMU. “Other” refers to countries that do not belong to any of the above-mentioned subregional economic groups. The Democratic Republic of the Congo, which is a member of both ECCAS and SADC is counted as ECCAS

### Remuneration of the health and care workforce accounts for an average 29% of country health spending, with marked differences between countries

**Fig. 1:**
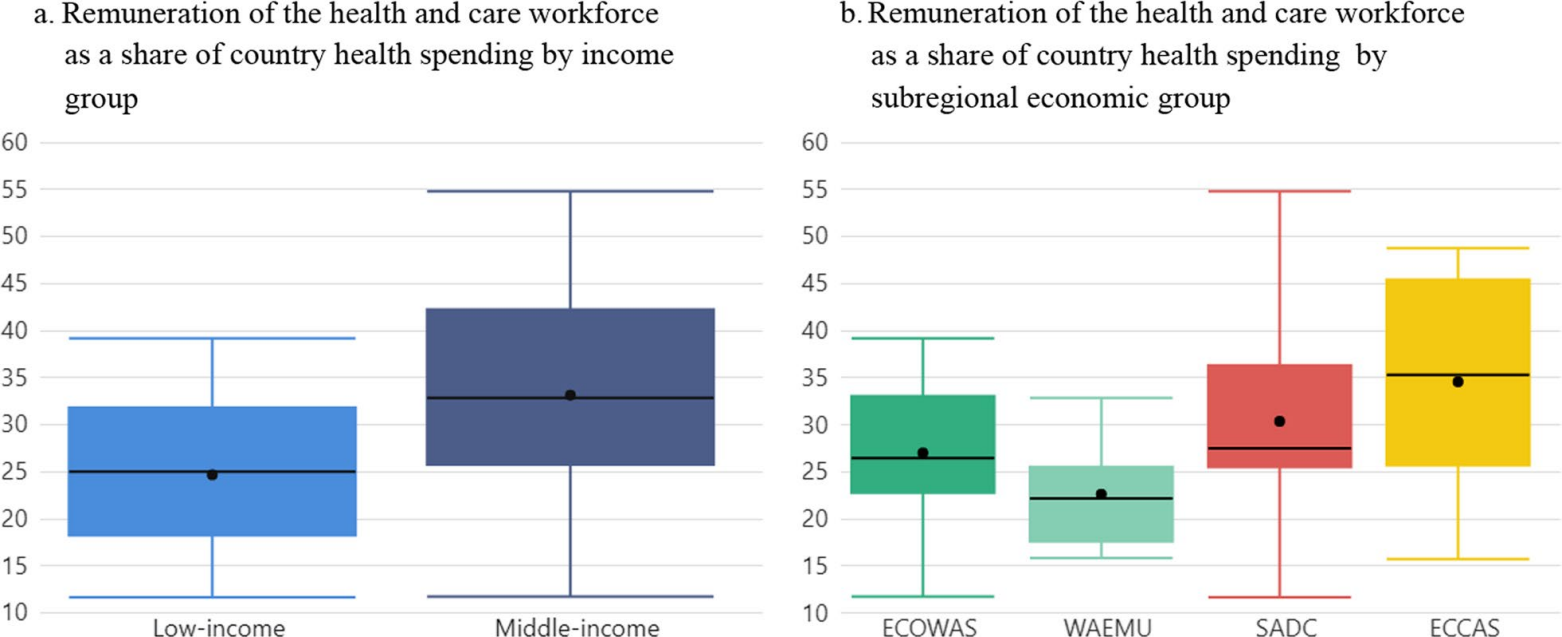
Health and care workforce remuneration as part of country health spending, by income and subregional economic group. Boxplots show the interquartile range (25th–75th percentile) of values. The vertical lines from the bars extend to the maximum and minimum values. The median is marked by the bar in the middle. The dot represents the average

Per capita health expenditure in these 33 countries averaged US$ 109 in real terms, equivalent to 10% of the average global per capita health expenditure (US$ 1140) and 85% of the average per capita expenditure in the 47 African countries (US$ 128).^10^ Average per capita HCW remuneration spending across these 33 countries amounted to US$ 38, 29% of health expenditure, from a minimal US$ 3 in Burundi to US$ 295 in South Africa—or one-sixth and one-half of overall health expenditure in these two countries, respectively. Furthermore, average HCW remuneration expenditure varies from one-quarter to one-third of health expenditure in the 16 low-income and 17 middle-income countries, respectively (Fig. 1a).

There are also differences across subregional economic groups (Fig. 1b). Average per capita HCW expenditure by subregional economic group is estimated at US$ 11, US$ 18, and US$ 94 in WAEMU, ECOWAS,^11^ and SADC countries, respectively (Appendix 2). On average HCW remuneration as a share of country health expenditure is the lowest in WAEMU countries, amounting to 23%—whereas for non-WAEMU ECOWAS countries and SADC countries this ratio is above 25%, averaging 27% and 30%, respectively. In ECCAS countries, HCW remuneration represents 35% of country health spending. Noteworthy is the higher variability in the SADC group compared to the other three (Fig. 1b).

### Government biggest contributor to HCW remuneration

**Fig. 2:**
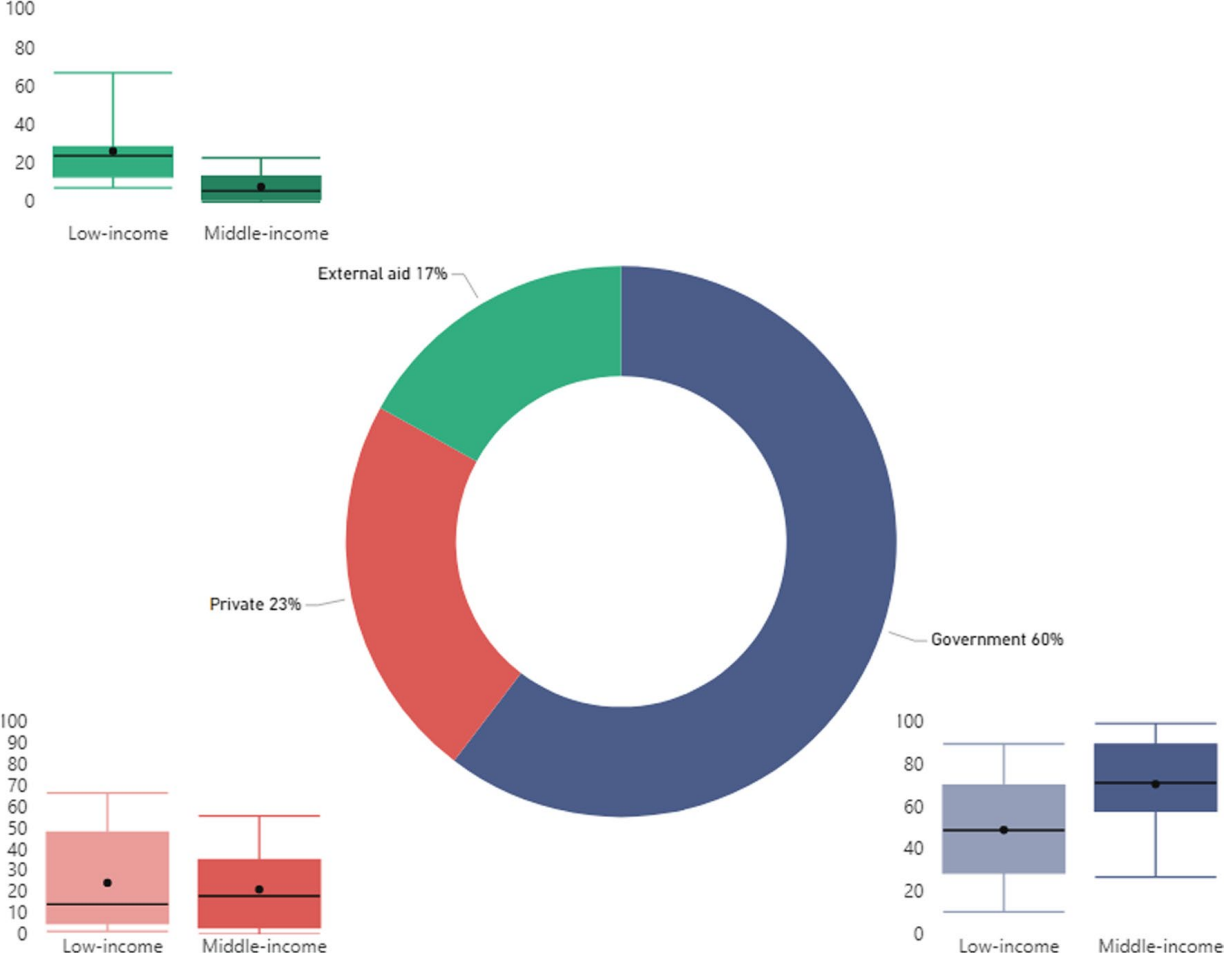
Funding sources for health and care workforce remuneration, across all countries and by income group. Boxplots show the interquartile range (25th–75th percentile) of values. The vertical lines from the bars extend to the maximum and minimum values. The median is marked by the bar in the middle. The dot represents the average

Government^12^ is the primary funding source for HCW remuneration, contributing to 60% on average. External aid and private sources contribute less, financing 17% and 23%, respectively. Nevertheless, by income group, contributions from each funding source vary significantly. In middle-income countries, governments fund 71%, compared to 49% in low-income countries. Conversely, external aid plays a critical role in in low-income countries, funding 27%—but in middle-income countries, it funds < 10%. Finally, private sources’ contributions are 24% and 21% in low- and middle-income countries, respectively (Fig. 2).

### More than half of government health spending goes to HCW remuneration

**Fig. 3:**
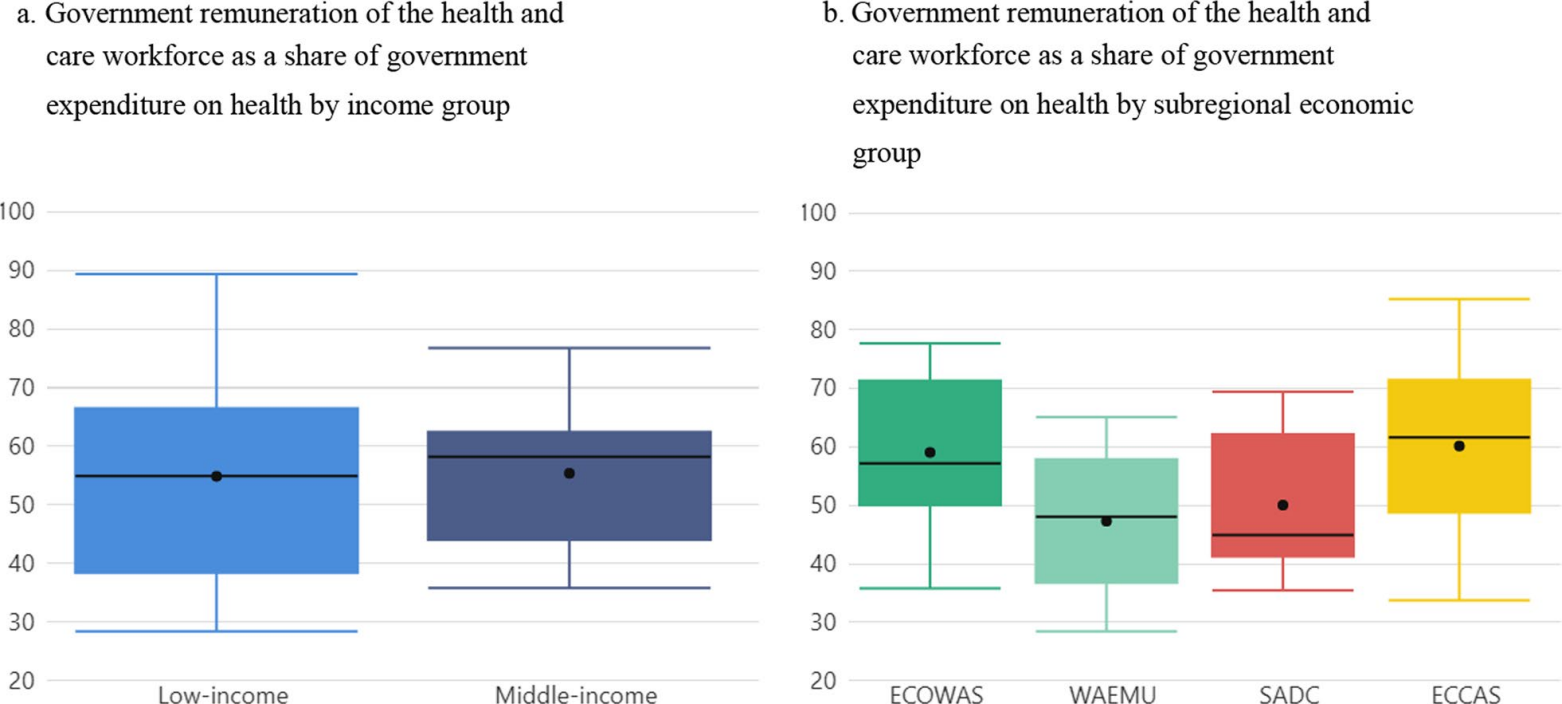
Government HCW remuneration as part of government health expenditure, by income and subregional economic group. Boxplots show the interquartile range (25th–75th percentile) of values. The vertical lines from the bars extend to the maximum and minimum values. The median is marked by the bar in the middle. The dot represents the average

Health and care workforce remuneration represents an average 55% of government spending on health. This is true for both low- and middle-income countries alike (Fig. 3a). However, there are marked differences between subregional economic groups. In ECOWAS and ECCAS countries, HCW remuneration accounts for 60% of government spending on health, compared with an average of 47% in WAEMU countries and 50% in SADC countries (Fig. 3b).

### Room for more government prioritization of HCW policies

**Fig. 4:**
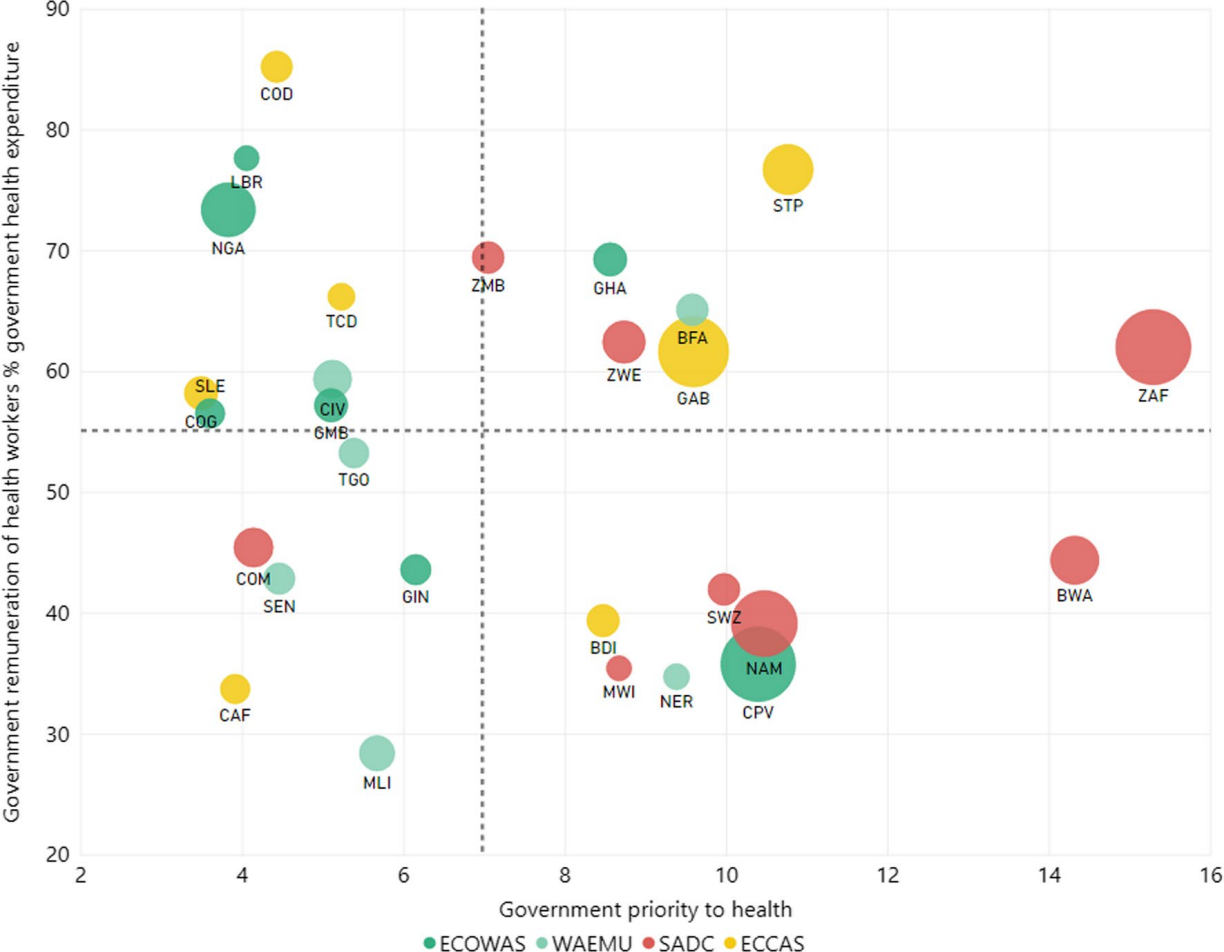
Government priority to health and part of government health spending on health and care workforce remuneration. Government priority to health refers to government expenditure on health as a share of government expenditure. The lines represent the average, across the 33 countries, of government priority to health (vertical line) and government remuneration of the health and care workforce as a share of government expenditure on health (horizontal line). Bubble size represents medical doctor density per 10,000 population

Looking at governments’ efforts, the SADC countries as a group placed a relatively higher government priority on health^13^ (see the red dots on the right side of Fig. 4). All but Comoros devoted to health more than the average 7% of general government spending calculated across the 33 countries, rising to 15% for South Africa. Moreover, in three countries—South Africa, Zambia, and Zimbabwe—government spending on HCW (see upper-right quadrant) is above-average; in these three countries, HCW remuneration accounts for > 55% of government health expenditure (above the horizontal line in Fig. 4). Whereas, in the other SADC countries, government spending on HCW remuneration was < 55%, below the average calculated across the 33 countries.

West African countries (green^14^ dots in Fig. 4), in contrast, spent less on health as a share of general government expenditure; all but four^15^ spent < 7%—the average of government spending on health calculated for the 33 countries (left side of Fig. 4)—but with varying levels of HCW remuneration spending as a share of government health spending. Some contributed > 55%, the average (upper-left quadrant); while in others like Mali, this figure is as low as 28% (lower-left quadrant). ECCAS countries showed no clear-cut pattern.

Finally, irrespective of their subregional economic group, countries on the right side of Fig. 4 presented a higher density HCW—as measured by the number of MDs per 10,000 inhabitants (see bubble size in Fig. 4). Nigeria, in the upper-left quadrant,^16^ is a noticeable exception.

## Discussion

### Findings

To our knowledge, this is the first attempt to provide a comparable HCW remuneration measurement for such a large number of African countries [20]. The estimates presented/discussed in this paper are based on country-produced data, not derived from modeling or a special data-collection effort but from actual data produced by country health accounts teams. Notably, 30 + African countries could produce data disaggregated enough using the international health spending measurement framework, despite the challenges/limitations of their respective underlying health information systems.

In terms of representativeness, all WAEMU countries but two—Benin and Guinea Bissau—are represented; the ECOWAS countries that are not WAEMU members are all included. This brings the total number of ECOWAS countries to 13 out of 15 (87%); and 50% of the SADC countries on the other hand.

Findings show that HCW remuneration spending is around 30% of country health expenditure. Governments are the main source of funding for HCW remuneration, contributing 60% on average. Furthermore, 55% of government health expenditure is spent on HCW remuneration, underscoring the complexity of the trade-offs to be made considering funding other inputs, e.g., pharmaceuticals. These results are difficult to compare with the only previous publication,^17^ which reported that HCW on average absorbs > 33% of government health expenditure, because our study does not include “capital” types of expenditure—namely, pre-service education, and does not include any high-income country [21].

The highest HCW spenders were those putting higher priority on health, mostly SADC countries—although this subregional economic group bore the highest heterogeneity, probably because of Comoros, and after excluding the Democratic Republic of the Congo, the two SADC countries with below-average priority on health. In fact, Comoros displayed below-average government spending on HCW remuneration, mirroring the West African countries’ spending pattern. A combination of different macroeconomic contexts as well different levels of HCW investment prioritization are likely to play a crucial role in explaining the different levels of spending for remuneration among countries.

Our results highlight the importance of government spending in funding HCW remuneration. The world economic outlook is currently uncertain, predicting a possible slowdown or weak economic growth. Within this context, maintaining or increasing the levels of public spending, and especially the one on HCW might become more of a challenge [22, 23]. Countries relying more on external sources could also face challenges, as it could generally be harder to maintain already-declining donor funding to low- and lower-middle-income countries’ health systems: external aid for health actually peaked in 2014 and has plateaued since [24]. It should be recognized, however, that lower levels of spending on HCW are likely to increase inequalities in access to service and may also be detrimental to the quality of care. Ten years ago, already, analyses determined a need for improved coordination between donor agencies and called for a paradigm shift towards investment in human resources, helping countries develop strategies reflecting specific domestic contexts and international best practices [4, 25]. Thus, mobilizing domestic resources and prioritizing HCW remuneration funding will be key, alongside policies aimed at investing in effective/efficient HCW policies [26].

### Limitations

While the current data set allows for general analysis of HCW remuneration, detailed analyses are not currently possible—e.g., comparison between health worker categories, gender pay gap, etc. It was not possible to assess the extent to which expenditures went to MDs, nurses, or other HCW, nor to determine the composition of their remuneration—salaries versus benefits, or policies like “pay for performance”—because the data set does not provide further breakdown by type of remuneration/payment. In addition, because there are currently no statistics that aggregate health workers into a single category, the results are expressed as a share of the country's health expenditure, not per health worker. Collecting systematic information on the private sector is also a serious challenge for several countries [27]. This is particularly the case for countries that do not have a national registry of health facility, which covers all private facilities, ideally by listing all facilities by level of care, from very peripheral first-contact health centers to high-end specialized hospitals, and including independent laboratories/pharmacies [28, 29].

When differentiating countries by risk/vulnerability status to further explore the potential impact on HCW remuneration, we noticed that two-thirds of the countries—11 fragile and 12 warning^18^—were in a “precarious” situation [30]. This impeded differentiating a pattern in levels spent on HCW remuneration. Finally, data availability and quality issues were also a limiting factor,^19^ and later we may have more countries as health accounts quality continues to improve [31, 32].

## Conclusions

The results clearly show that the remuneration of the health and care workforce is an important part of government health spending, with an average of 55% of government health spending devoted to it—with some countries well below or above the average. Health prioritization and the macroeconomic context are certainly two elements that play a key role in explaining differences between countries, and would need to be better integrated in further analysis of HCW remuneration spending, especially when considering an increase in the health and care workforce and its impact on remuneration-related expenditures. This is particularly relevant as the 10-million shortage by 2030 will be concentrated in Africa. WHO has called upon governments and partners in countries with greatest health workforce shortages—majority of which part of our study—to take action to double their workforce by 2030. While HCW remuneration accounts for half government health spending, doubling the health and care workforce will require significant increases in government health spending. It will be important to consider options within the fiscal space to allow a sustainable financial growth for the education and employment of health workers, alongside the optimization of the health and care workforce and a focus on efficiencies.

Furthermore, while the results provide some insight into the magnitude of HCW remuneration, more work and research is needed to obtain a more disaggregated and detailed picture, particularly with regard to remuneration across health occupations, between genders, and between the public and private sectors—e.g., private sources’ size would need confirmation in the future. At present, however, HCW remuneration expenditure—albeit part of country-produced health accounts—has not made into the GHED, as harmonization of concepts, collaboration with national counterparts, and integration of feedback require more time/resources. It is a work in progress, and this type of expenditure will eventually make it to publication in the coming years. This is part of a sustained effort by WHO and its partners to provide by-topic detailed thematic guidance, in-country technical assistance, and data-quality assurance to Member States. For example, for a subset of countries, disaggregated data on diseases and health programs—e.g., spending on family planning and primary health care—have been published for the past 5 years.

Capturing trends in the amounts spent on HCW remuneration, including by source of funding, will provide better insights for developing policies related to the funding of HCW remuneration, thereby helping to shape the supply for health workers. Systematically comparing the components of HCW expenditure across countries will allow better identification of different funding sources—either stable or volatile in nature—and their impact on HCW investment over time. Such stocktaking is important for countries, WHO, and other relevant agencies to inform necessary policy changes. Moreover, beyond country comparisons, such as ours, countries interested in further analysis could conduct deep dive analysis, looking at specific variables, to track policy questions of interest—e.g., on recruitment/retention and/or assessing government spending on HCW relative to total government spending on human resource from general government spending.

## Data Availability

The data sets analyzed during the current study are available from https://www.dropbox.com/s/grdu990bqimhxwq/hwf_data_final_20230220.xlsx?dl=0.

## Appendix 1: Country list

**Table:**

Country name	Country code	Income group^a^	Subregional economic group	Latest available year
Botswana	BWA	Middle-income	SADC	2017
Burkina Faso	BFA	Low-income	WAEMU	2019
Burundi	BDI	Low-income	ECCAS	2018
Cabo Verde, Republic of	CPV	Middle-income	ECOWAS	2016
Central African Republic	CAF	Low-income	ECCAS	2018
Chad	TCD	Low-income	ECCAS	2018
Comoros	COM	Middle-income	SADC	2019
Congo	COG	Middle-income	ECCAS	2018
Côte d'Ivoire	CIV	Middle-income	WAEMU	2018
Democratic Republic of the Congo	COD	Low-income	ECCAS	2019
Eswatini	SWZ	Middle-income	SADC	2017
Ethiopia	ETH	Low-income	Other	2017
Gabon	GAB	Middle-income	ECCAS	2019
Gambia	GMB	Low-income	ECOWAS	2015
Ghana	GHA	Middle-income	ECOWAS	2015
Guinea	GIN	Low-income	ECOWAS	2019
Kenya	KEN	Middle-income	Other	2019
Liberia	LBR	Low-income	ECOWAS	2019
Malawi	MWI	Low-income	SADC	2018
Mali	MLI	Low-income	WAEMU	2018
Mauritania	MRT	Middle-income	Other	2017
Namibia	NAM	Middle-income	SADC	2016
Niger	NER	Low-income	WAEMU	2019
Nigeria	NGA	Middle-income	ECOWAS	2019
Sao Tome and Principe	STP	Middle-income	ECCAS	2017
Senegal	SEN	Middle-income	WAEMU	2016
Sierra Leone	SLE	Low-income	ECOWAS	2018
South Africa	ZAF	Middle-income	SADC	2016
South Sudan	SSD	Low-income	Other	2017
Togo	TGO	Low-income	WAEMU	2019
Uganda	UGA	Low-income	Other	2019
Zambia	ZMB	Middle-income	SADC	2016
Zimbabwe	ZWE	Middle-income	SADC	2018

ECOWAS excludes countries that are also members of WAEMU. “Other” refers to countries that do not belong to any of the subregional groups mentioned (SADC, WAEMU, ECOWAS, and ECCAS). Middle-income regroups lower middle and upper middle income groups into one

^a^World Bank 2019 grouping, as this is the last pre-pandemic data

## Appendix 2: Expenditure on the health and care workforce remuneration

See Tables 3, 4, 5

**Table 3:** Per capita expenditure on health, health and care workforce (HCW) remuneration, and health and care workforce remuneration as a share of country health expenditure

Statistics	Per capita health expenditure (US$)	Per capita expenditure on HCW remuneration (US$)	Expenditure on HCW remuneration as a share of country health expenditure (%)
Minimum	20.6	3.4	11.7
Maximum	538.8	295.2	54.8
Average	108.8	37.6	29.1
Median	52.6	14.1	27.0

**Table 4:** Average expenditure on the health workforce (HCW) remuneration, per capita and as a share of country health expenditure, by income group

Income group	Per capita expenditure on HCW remuneration (US$)	Expenditure on HCW remuneration as a share of country health expenditure (%)
Low-income	9	25
Middle-income	65	33
Total	38	29

The income groups are as per the World Bank 2019 classification of income groups; lower-middle–income and upper-middle–income groups are merged into a single middle-income group

**Table 5:** Average expenditure on remunerating the health and care workforce (HCW) remuneration, per capita and as a share of country health expenditure, by subregional economic group

Subregional economic group	Per capita expenditure on HCW remuneration (US$)	Expenditure on HCW remuneration as a share of country health expenditure (%)
ECOWAS (non-WAEMU)	18	27
WAEMU	11	23
ECOWAS	15	25
ECCAS	31	35
SADC	94	30
Other	15	30
Total	38	29

ECCAS, ECOWAS, WAEMU, and SADC, respectively, stand for the Economic Community of Central African States, the Economic Community of West African States, the West African Economic and Monetary Union, and the Southern African Development Community. “Other” refers to countries that do not belong to any of the above-mentioned subregional economic groups. ECOWAS (non-WAEMU) excludes countries that are also members of WAEMU

## Abbreviations

COVID-19: Severe acute respiratory syndrome-related coronavirus 2, or SARS-CoV-2
ECCAS: Economic Community of Central African States
ECOWAS: Economic Community of West African States
FP: Factor of provision
GHED: WHO’s Global Health Expenditure Database
HCW: Health and care workforce
MD: Medical doctor
NHWA: WHO’s National Health Workforce Accounts database
SADC: Southern African Development Community
SDG: United Nations Sustainable Development Goal
SHA: System of health accounts
THE: Total health expenditure
UHC: Universal health coverage
WAEMU: West African Economic and Monetary Union
WHO: World Health Organization

## Footnotes

1 All of the values in this paper are per year. This can be either calendar or fiscal, depending on the country's context.

2 “Public” means government-owned resources (collected through taxation) and mandatory contributions to social health insurance schemes, when existing. “Private” refers to out-of-pocket payments and contributions to voluntary health insurance, i.a. “External” refers to funds from abroad (e.g., foreign countries, multilateral agencies).

3 World Health Organization (WHO). The Global Health Expenditure Database [Internet]. Geneva: WHO; 2023. Available from: https://apps.who.int/nha/database/Home/Index/en (accessed 26 Sep 2023).

4 World Health Organization (WHO). Methodology for the update of the global health expenditure database 2020–2022: technical note [Internet]. Geneva: WHO; 2023. Available from: https://iris.who.int/handle/10665/365909 (accessed 26 Sep 2023).

5 World Health Organization (WHO). Global spending on health 2020: Weathering the storm [Internet]. Geneva: WHO; 2020. Available from: https://apps.who.int/iris/handle/10665/337859 (accessed 26 Sep 2023).

6 World Health Organization (WHO). Global spending on health: rising to the pandemic’s challenges [Internet]. Geneva: WHO; 2022. Available from: https://www.who.int/publications/i/item/9789240064911 (accessed 26 Sep 2023).

7 Middle-income countries include lower-middle and upper-middle–income countries, as per the World Bank 2019 classification of income groups. A 34^th^ country, Seychelles, is not included as it was the only high-income country with available disaggregated spending amounts on HCW expenditure.

8 The Democratic Republic of the Congo, which is a member of both ECCAS and SADC, is counted as ECCAS.

9 Throughout the article, unless otherwise indicated, ECOWAS excludes WAEMU member countries.

10 The reference year for global and regional average per capita health expenditure is 2019, using the 2021 GHED update.

11 US$ 15 across the 13 ECOWAS countries with disaggregated data available; WAEMU countries included.

12 “Government” includes funding sources from government resources (general taxation) and contributions to mandatory health insurance schemes. “External aid” refers to development partner funds handled by non-profit entities or channeled through governments. “Private sources” refer to domestic funds, e.g., out-of-pocket payments and contributions to voluntary health insurance schemes, i.a.

13 Proxied by government expenditure on health as a share of general government expenditure, as on the x axis of Fig. 4.

14 Dark and light green, respectively, for ECOWAS and WAEMU countries.

15 Burkina Faso, Cabo Verde, Ghana, and Niger.

16 High expenditure on HCW remuneration as a share of government expenditure on health, but with relatively lower government priority on health (government expenditure on health as a share of general government expenditure).

17 Though, in their paper “Paying for needed health workers for the SDGs: an analysis of fiscal and financial space” In: Health employment and economic growth: an evidence base. Geneva: World Health Organization, 2017, also available, Lauer et al. made a crude attempt to express wage bill as a proportion of public spending on health for 136 countries, and their estimate was 57%.

18 **Stable countries**: Botswana, Cabo Verde, Gabon, Ghana, and Namibia. **Warning countries**: Côte d’Ivoire, Comoros, Gambia, Eswatini, Liberia, Malawi, São Tomé and Príncipe, Senegal, Sierra Leone, South Africa, Togo, and Zambia. **Alert countries**: Burkina Faso, Burundi, Chad, Central African Republic, Democratic Republic of the Congo, Guinea, Mali, Niger, Nigeria, and Zimbabwe. Five other countries remain unclassified.

19 One country with available HCW-disaggregated expenditure data could not be included here because more than 30% of its health expenditure was “unclassified:” for > 30% of this country’s expenditure, it was unclear whether it was on HCW, pharmaceuticals, or other type of inputs—potentially underestimating the HCW amounts reported.
